# Risk of Psychosis in People of Irish and Chinese Ethnicities in Yorkshire

**DOI:** 10.1192/bjo.2025.10233

**Published:** 2025-06-20

**Authors:** Sarthak Sinha, Tariq Mahmood, Alastair Cardno, Emi Obode

**Affiliations:** 1 University of Leeds, Leeds, United Kingdom; 2 St James University Hospital, Leeds, United Kingdom

## Abstract

**Aims:** Studies have shown an elevated risk of psychosis among migrants and ethnic minorities, but there has been little investigation of risks for Irish and Chinese ethnic groups in the UK. The aim of this study is to investigate the risk of first-episode psychosis in White Irish and Chinese ethnic groups compared with the White British population in West Yorkshire.

**Methods:** Data from local census and two Early Intervention in Psychosis services for individuals aged 15–34 with first episode psychosis between 2013–2015 was collected. Risk ratios for combined locations were calculated using Mantel–Haenszel fixed effects models.

**Results:** The White Irish group showed a non-significant but consistent trend of around a 2-fold elevated risk of first episode psychosis (RR 2.27, 95% CI 0.95 to 5.46). The Chinese group did not show a significantly elevated risk (RR 0.4, 95% CI 0.13 to 1.25).

**Conclusion:** Although not statistically significant, the study suggests a consistent trend of elevated psychosis risk in the White Irish group. Further research is needed to validate these findings and determine key contributing factors.

